# Zerumbone Attenuates the Severity of Acute Necrotizing Pancreatitis and Pancreatitis-Induced Hepatic Injury

**DOI:** 10.1155/2012/156507

**Published:** 2012-03-05

**Authors:** Deng Wenhong, Yu Jia, Wang Weixing, Chen Xiaoyan, Chen Chen, Xu Sheng, Jin Hao

**Affiliations:** ^1^Department of General Surgery, Renmin Hospital of Wuhan University, Hubei 430060, China; ^2^Department of General Surgery, People's Hospital of Guangxi Zhuang Autonomous Region, Nanning 530000, China

## Abstract

This paper investigated the potential effects of zerumbone pretreatment on an acute necrotizing pancreatitis rat model induced by sodium taurocholate. The pancreatitis injury was evaluated by serum AMY, sPLA2, and pancreatic pathological score. Pancreatitis-induced hepatic injury was measured by ALT, AST, and hepatic histopathology. The expression of I-**κ**B**α** and NF-**κ**B protein was evaluated by western blot and immunohistochemistry assay while ICAM-1 and IL-1**β** mRNA were examined by RT-PCR. The results showed that AMY, sPLA2, ALT, and AST levels and histopathological assay of pancreatic and hepatic tissues were significantly reduced following administration of zerumbone. Applying zerumbone also has been shown to inhibit NF-**κ**B protein and downregulation of ICAM-1 and IL-1**β** mRNA. The present paper suggests that treatment of zerumbone on rat attenuates the severity of acute necrotizing pancreatitis and pancreatitis-induced hepatic injury, via inhibiting NF-**κ**B activation and downregulating the expression of ICAM-1 and IL-1**β**.

## 1. Introduction

Acute necrotizing pancreatitis (ANP) is an acute abdomen-characterized disease, and it is characterized by elevated pancreatic enzyme levels. Published data has indicated that ANP is an inflammatory disease with a systemic inflammatory response syndrome and a multiple organ dysfunction [1, 2], and hepatic injury is a manifestation of systemic inflammatory response during ANP [3]. Hepatic microcirculatory dysfunction, tissue hypoxia, and inflammatory cytokines also induce hepatic injury by resident macrophages during the progress of pancreatitis [4–6]. Therefore, an effective pharmacological amelioration of hepatic injury could improve the quality of life in ANP patients.

Zerumbone (2,6,9,9-tetramethylcycloundeca-2,6,10-trien-1-one), as the main component of the essential oil of Zingiber zerumbet, is a monocyclic sesquiterpene containing a cross-conjugated dienone system [7]. It has been shown to have antineoplasms and anti-inflammatory properties in animal models. Some studies reported that zerumbone had the ability to suppress the proliferation of cancer cells and inhibit the invasion of tumor in breast, pancreas, colon, lung, and skin [8–11]. The anti-inflammation effect of zerumbone was evaluated by the suppression of proinflammatory gene and the antioxidant response of element-dependent detoxification pathway [12–17]. Moreover, Annamaria et al. indicated that zerumbone exerted a beneficial effect on inflammatory parameters following experimental pancreatitis [18]. However, there is little available information on how zerumbone regulates pancreatitis-induced hepatic injury. This work places emphasis on the effect of zerumbone on hepatic injury following ANP, suggesting that zerumbone could be a good candidate to attenuate the severity of acute necrotizing pancreatitis and pancreatitis-induced hepatic injury.

## 2. Materials and Methods

### 2.1. Animals and Reagents

Male SPF Wistar rats, weighing 200 to 250 g, were obtained from the Center of Experimental Animals of Hubei Academy of Medical Sciences, Wuhan, China. The animals were kept at room temperature and 12 h light-dark cycles, and with free access to water. Rats in this study were maintained in accordance with the principles of the 1983 Declaration of Helsinki by the Ethics Committee of Wuhan University. Zerumbone (purity > 99%) was obtained from Kingherb's company (Hainan, China). Sodium taurocholate and dimethyl sulfoxide (DMSO) were purchased from Sigma Aldrich Company (St. Louis, MO). Primers were designed and synthesized by Invitrogen Corporation (Carlsbad, CA).

### 2.2. Induction of Acute Necrotizing Pancreatitis

Rats were fasted overnight and given fresh tap water ad libitum. Anesthesia was administered by intraperitoneal injection of 10% chloraldurat (3 mL/kg). The pancreatic bile duct was cannulated through the duodenum. Acute necrotizing pancreatitis was induced by a standardized retrograde infusion of a freshly prepared 5% sodium taurocholate solution (1 mg/kg) into the biliary-pancreatic duct. Isotonic saline solution (20 mL/kg) was injected into the back to compensate for fluid loss.

### 2.3. Experimental Design

The primary objective of a preliminary study was to obtain the optimal dose of zerumbone for preventing pancreatitis-induced hepatic injury. The second study was a formal study, which was to explore the effect of zerumbone on hepatic injury following ANP. Zerumbone was dissolved in vehicle (10% DMSO v/v) in this study.

In the preliminary study, rats were randomly divided into sham-operated group, acute necrotizing pancreatitis group, and zerumbone-pretreated subgroups (*n* = 8 per group) (Table 1). Zerumbone was injected via femoral vein at various doses (5, 10, 20, and 40 mg/kg) in zerumbone pretreated group rats. After half an hour of injecting, acute necrotizing pancreatitis was induced by retrograde infusion 5% sodium taurocholate solution. In sham-operated group, infusion isotonic saline solution instead of taurocholate. All rats were sacrificed at 12 h after induction of pancreatitis because intrapancreatic damage reached a peak [20]. The effect of zerumbone was evaluated by the levels of serum amylase (AMY), secretory phospholipase A_2_ (sPLA_2_), and alanine aminotransferase (ALT), which had been described before [18].

In the formal study, 10 mg/kg zerumbone was adopted as the optimal dose. 160 male Wistar rats were randomly divided into four groups: (1) taurocholate + vehicle group (TC group, *n* = 64); (2) saline + vehicle as the control group (CON group, *n* = 64); (3) taurocholate + zerumbone group (ZP group, *n* = 16); (4) saline + zerumbone group (Z group, *n* = 16). In the TC group, rats received vehicle (10% DMSO, 2 mL/kg) via femoral vein, half an hour prior to 5% sodium taurocholate infusion. In the CON group, rats only received the vehicle. In the ZP group, rats received 10 mg/kg zerumbone dissolved in the vehicle (10% DMSO v/v). In the Z group, rats received the zerumbone and saline (Table 2). In the ZP and Z groups, rats were only at the time point of 12 h (*n* = 16). Furthermore, in the TC and CON groups, rats were subdivided into subgroups of 1, 3, 6, and 12 h (*n* = 16) (Table 2).

For each group of the two studies, rats were sacrificed by taking blood via heart puncture. Blood samples were collected for centrifuging, and serum was stored at −20°C. After sacrifice, the head of the pancreatic tissue and the right lobe of hepatic tissue were harvested and fixed in 4% PBS-buffered formaldehyde for histopathology observation. The remaining part of the pancreatic and hepatic tissues were immediately snap frozen in liquid nitrogen and stored at −80°C for assay.

### 2.4. Enzyme Assay

Plasma amylase (AMY), alanine aminotransferase (ALT), and aspartate transaminase (AST) were measured by an automatic biochemistry analyzer with standard techniques (Olympus Optical Ltd., Japan). Serum sPLA_2_ activity was measured by sPLA_2_ Assay Kit according to the manufacturer's instructions (Ann Arbor, MI).

### 2.5. Histopathological Examination

Pancreatic and hepatic tissues were fixed, subjected to conventional processing and sectioning, followed by hematoxylin-eosin (H&E) staining. The sections were evaluated by two pathologists who were blinded to this study. Pancreatic histological assessment was determined by edema, inflammation, hemorrhage, and necrosis according to the scale described by Schmidt et al. [19]. The severity of hepatic injury was determined by a point-counting method description by Camargo et al. [21].

### 2.6. Immunohistochemisty Assay

Pancreatic-tissue sections (4 *μ*m) were obtained from paraffin-embedded tissues. Under deparaffinization, 0.3% (v/v) hydrogen peroxide was used to inactivate endogenous peroxidase activity. The sections underwent a blocking step with 5% normal goat serum diluted in PBS. Endogenous biotin and avidin binding sites were blocked by avidin and biotin, respectively. Sections were incubated with rabbit polyclonal anti-rat NF-*κ*B p65 antibody (1 : 200, Sigma, St. Louis, MO) in a moisture chamber. Sections were then counterstained with hematoxylin. Negative control studies were performed in which PBS was used instead of primary antibody.

### 2.7. Western Blot

Proteins (including cytoplasmic and nuclear proteins) were extracted by the Nuclear-Cytosol Extraction Kit (Applygen Technologies Inc., Beijing, China), followed by manufacturer's instructions. Proteins were evaluated by the Bradford method with bovine serum albumin as a standard. 40 mg protein samples were electrophoresed in 8% sodium dodecylsulphate polyacrylamide (SDS-PAGE) gels and transferred to nitrocellulose membranes. Membranes were blocked with blocking buffer (TBS containing 5% nonfat dry milk, 0.1% Tween-20) for 2 h at room temperature. The cytoplasmic proteins were incubated with primary antibodies of rabbit polyclonal anti-rat I-*κ*B*α* antibody (1 : 1000, Santa Cruz, CA), actin antibody (1 : 2000, Santa Cruz, CA). Meanwhile, the nuclear proteins were incubated with rabbit polyclonal anti-rat NF-*κ*B p65 antibody (1 : 2000, Sigma, St. Louis, MO) and actin antibody (1 : 2000, Santa Cruz, CA) overnight at 4°C. The membrane was washed with TBST (TBS containing 0.05% Tween-20) and then incubated with horseradish peroxidase-conjugated goat anti-rabbit secondary antibodies (1 : 5000, Pierce Biotechnology, Rockford, IL) for 1 h at room temperature. After repeated washings with TBST, the antibody-antigen complexes were detected by ECL reagent (Immobilon Western HRP Substrate, Millipore Corporation, Bedford, MA).

### 2.8. Reverse Transcriptase Polymerase Chain Reaction (RT-PCR)

Total RNA was extracted from one hundred milligram frozen pancreatic and hepatic tissues. RNA was reverse-transcribed to complementary DNA according to the manufacturer's instructions of Revert Aid First Strand cDNA Synthesis Kit (Fermentas, Hanover, MD). Polymerase chain reaction was performed with the primers for intercellular adhesion molecule-1 (ICAM-1) (F: CGGTAGACACAAGCAAGAGA; R: GCAGGGATTGACCATAATTT; 517 bp; NM-012967); interleukin-1*β* (IL-1*β*) (F: CCAGGATGAGGACCCAAGCA; R: TCCCGACCATTGCTGTTTCC; 519 bp; NM-031512); glyceraldehyde-3-phosphate dehydrogenase (GAPDH) (F: TCATGAAGTGTGACGTGGACATC; R: CAGGAGGAGCAATGATCTTGATCT; 309 bp; NM-017008). PCR was performed by using a Gene Cycler (Bio-Rad, Hercules, CA). Amplification steps were ICAM-1 (initial denaturation 94°C 5 min, then denaturation 94°C 30 sec, annealing 56°C 30 sec, extension 72°C 1 min for 35 cycles, final extension 72°C 7 min); IL-1*β* (initial denaturation 94°C 5 min, then denaturation 95°C 30 sec, annealing 63°C 45 sec, extension 72°C 35 sec for 35 cycles, final extension 72°C 7 min); GAPDH (initial denaturation 94°C 5 min, then denaturation 94°C 30 sec, annealing 55°C 30 sec, extension 72°C 80 sec for 35 cycles, final extension 72°C 7 min). PCR products were electrophoresed in 2% agarose gel containing ethidium bromide (0.5 mg/mL). Gels were under UV light and then photographed. Band intensity was determined by optical density with PCR product/GAPDH ratios.

### 2.9. Statistical Analysis

All data were expressed as means ± standard deviation values. Data were compared between all groups by one-way analysis of variance (ANOVA) except hepatic histopathological examination, which was analyzed by Kruskall-Wallis nonparametric test, followed by Mann-Whitney test. Statistical analysis was performed with the SPSS statistical package (SPSS 13.0 for Windows; SPSS Inc., Chicago, IL). A value of *P* < 0.05 was regarded as a significant difference.

## 3. Results

### 3.1. Preliminary Study Results

The results indicated that neither 5 mg/kg nor 40 mg/kg zerumbone improve serum sPLA_2_ and ALT levels. Compared to 20 mg/kg, concentration of 10 mg/kg has already shown significant reduction of sPLA_2_ and ALT levels (*P* < 0.05) (Table 3).

### 3.2. Analysis of Serum AMY, sPLA_2_, ALT, and AST

**Figure 1:**
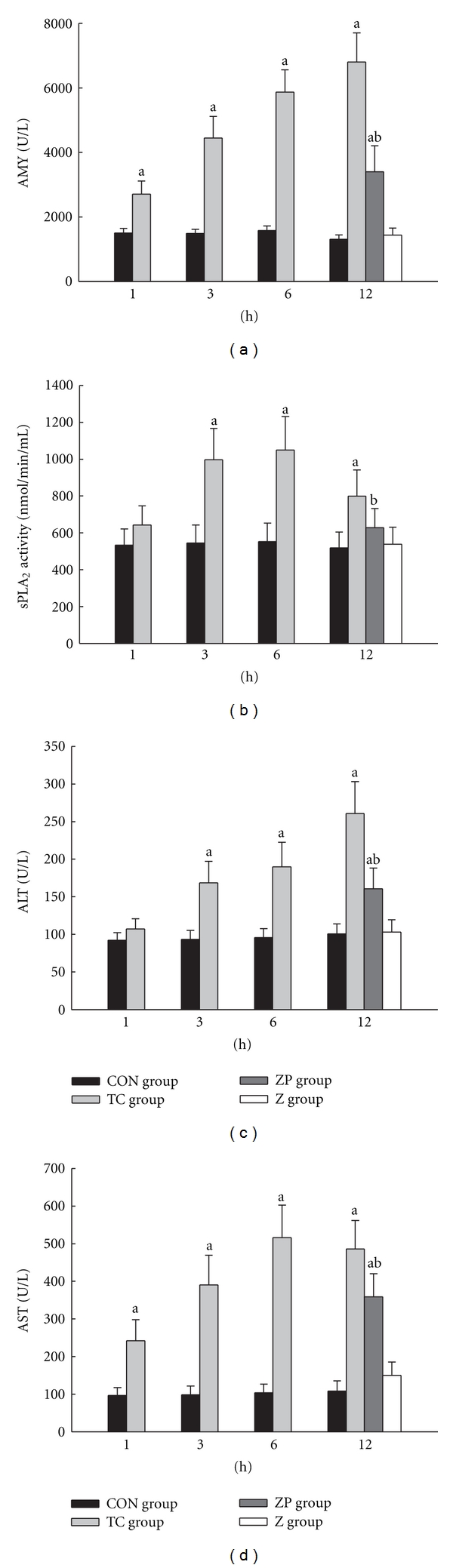
(a) Levels of serum amylase (AMY), (c) alanine aminotransferase (ALT), and (d) glutamic oxalacetic transaminase (AST) were measured by an automatic biochemistry analyzer with standard techniques (Olympus Optical Ltd., Japan); (b) serum secretory phospholipase A_2_ (sPLA_2_) activity was measured by sPLA_2_ Assay Kit according to the manufacturer's instructions.  ^a^ *P* < 0.05 versus CON group.  ^b^ *P* < 0.05 versus TC group.

Compared with the CON group, the TC group had significant increased serum AMY, sPLA_2_, ALT, and AST levels from 1 h to 12 h (*P* < 0.05) except for 1 h sPLA_2_ and ALT. In the ZP group, pretreatment with zerumbone significantly reduced serum enzyme levels compared with the TC group (*P* < 0.05). In Z group, all serum enzyme levels were not significant compared with the CON group (Figures 1(a)–1(d)).

### 3.3. Histopathological Assay

**Figure 2:**
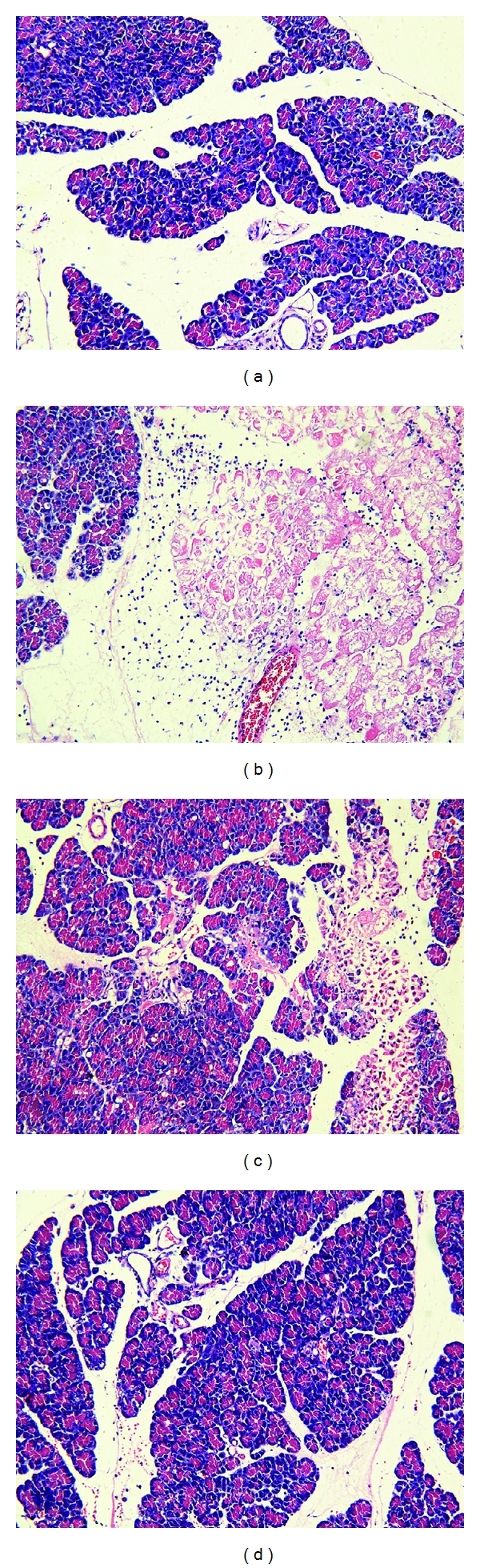
Morphologic changes in pancreatic tissue at 12 h. Representative hematoxylin/eosin-stained sections were examined by light microscopy (original magnification: 200x). (a) The CON group, (b) The TC group, (c) The ZP group, and (d) The Z group.

Representative pathological changes in pancreatic tissue were shown in Figures 2(a)–2(d). Injuries were estimated by Schmidt's description including edema, inflammation, hemorrhage, and necrosis [19]. The pancreatic microscopy was similar among the CON subgroups exhibiting no hemorrhage or necrosis. In the TC group, 5% sodium taurocholate promoted the destruction of acinar cell. However, pretreatment with zerumbone decreased the pancreatic histopathological score clearly in the ZP group (Figure 4).

**Figure 3:**
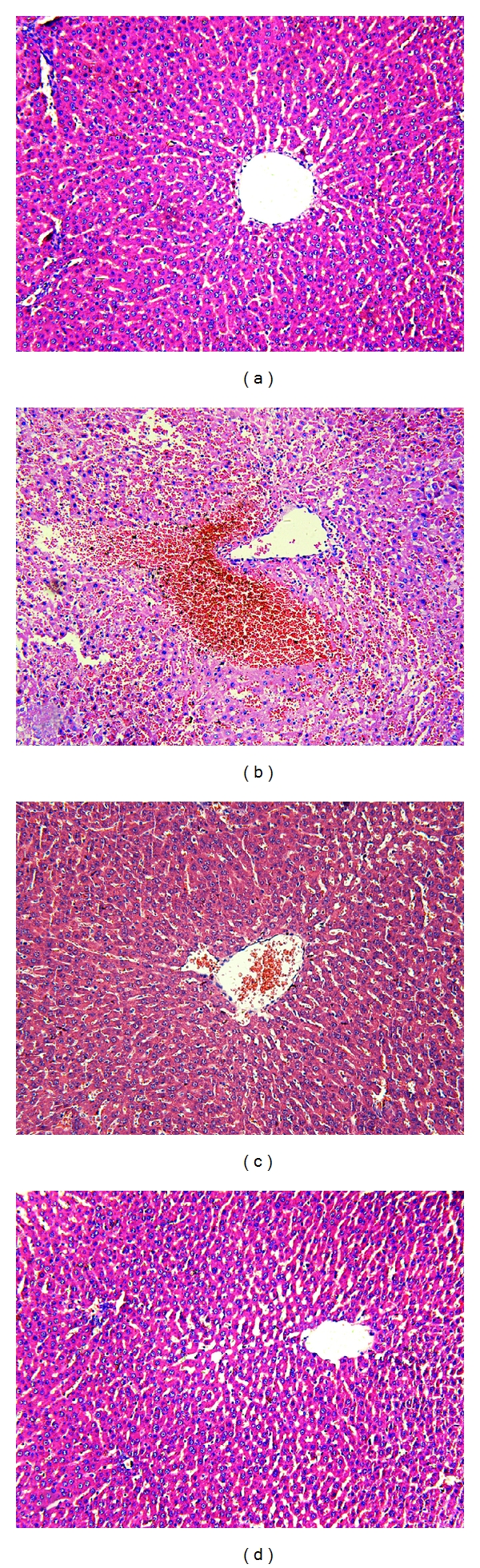
Morphologic changes in hepatic injury at 12 h. Representative hematoxylin/eosin-stained sections were examined by light microscopy (original magnification: 200x). (a) The CON group, (b) The TC group, (c) The ZP group, and (d) The Z group.

**Figure 4:**
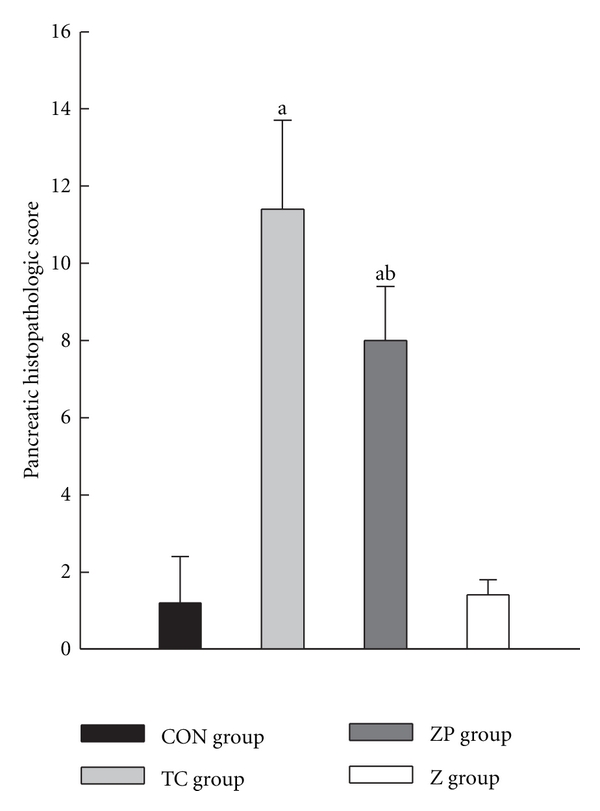
Pancreatic histopathological scores in all groups. The pancreatic tissue was with hematoxylin-eosin staining. The sections were evaluated by two independent pathologists who were blinded to this research. Time course changes of the pancreatic histological assessment were scored based on edema, inflammation, hemorrhage, and necrosis according to the scale described by Schmidt et al. [19]. ^a^ *P* < 0.05 versus CON group. ^b^ *P* < 0.05 versus TC group.

The change in hepatic sections was determined by Camargo's description [21]. There were four grades: grade 0: minimal or no evidence of injury; grade I: mild injury consisting in cytoplasmic vacuolation and focal nuclear pyknosis; grade II: moderate to severe injury with extensive nuclear pyknosis, cytoplasmic hypereosinophilia, and loss of intercellular borders; grade III: severe necrosis with disintegration of hepatic cords, hemorrhage, and neutrophil infiltration. Pathological changes in hepatic tissue were shown in Figures 3(a)–3(d). In the TC group, 5% sodium taurocholate had destroyed most of the liver cells and caused obvious bleeding. However, the hepatic pathological grade reduced to a much lower level by pretreatment with zerumbone in the ZP group (Table 4).

### 3.4. Immunohistochemisty Analysis

**Figure 5:**
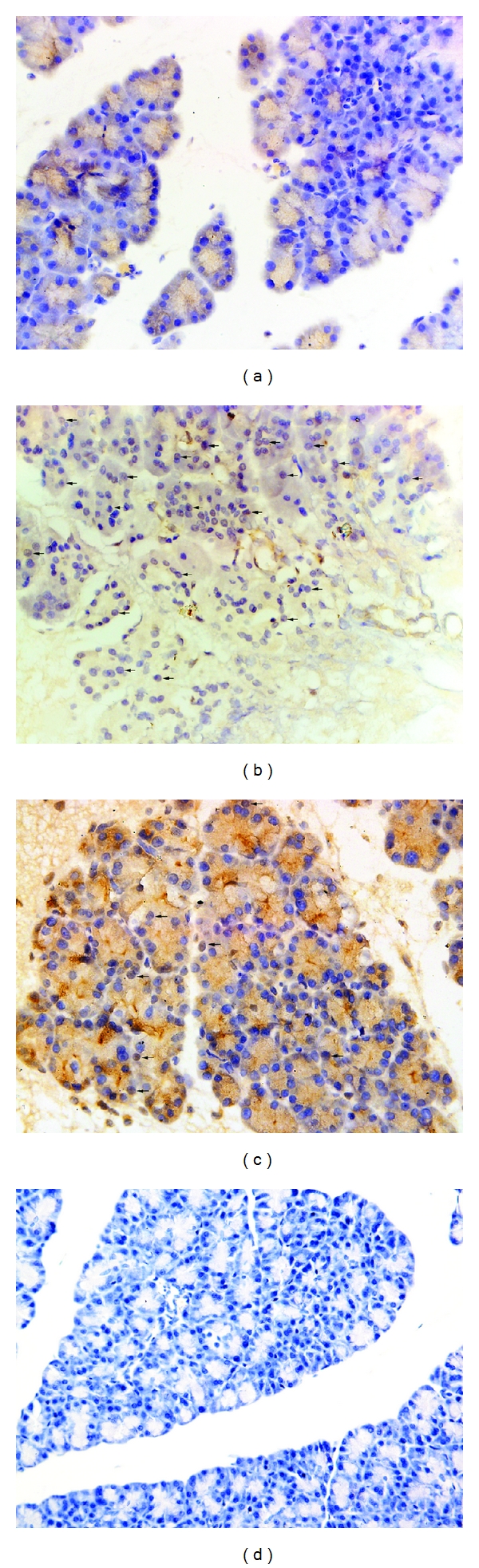
Cellular localization of NF-*κ*B p65 protein in pancreas in different groups (original magnification: 400x). (a) In the CON group, weak immunoreactivity mainly in the cytoplasm. (b) In the TC group, intense immunoreactivity in the nucleus. (c) In the ZP group, low immunoreactivity in the nucleus. (d) Negative control.

**Figure 6:**
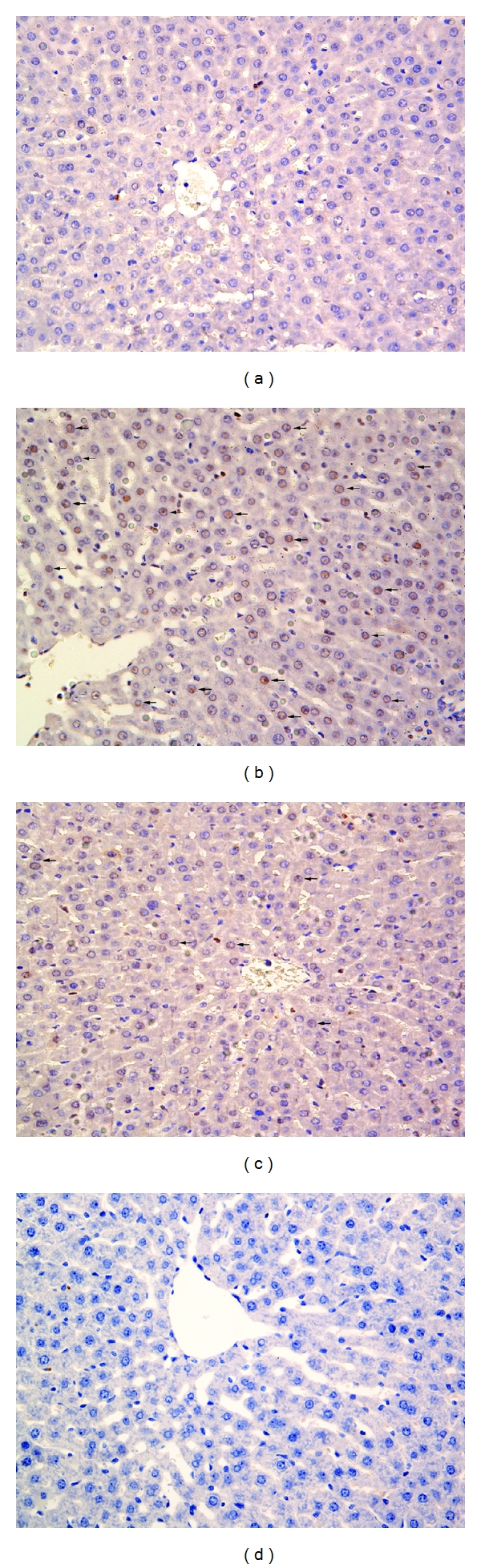
Cellular localization of NF-*κ*B p65 protein in hepatic tissue in different groups (original magnification: 400x). (a) In the CON group, weak immunoreactivity mainly in the cytoplasm. (b) In the TC group, intense immunoreactivity in the nucleus. (c) In the ZP group, low immunoreactivity in the nucleus. (d) Negative control.

In performing the localization of NF-*κ*B expression, immunohistochemical assay was used. There were no significant changes in the immunoreactivity of NF-*κ*B in the CON group. NF-*κ*B expression was expressed mainly in the cytoplasm in pancreatic or hepatic tissue (Figures 5(a), 6(a)). Following pancreatitis, NF-*κ*B immunoreactivity highly expresssed in nucleus (Figures 5(b), 6(b)). However, a marked decrease in NF-*κ*B staining was found in the nucleus with the zerumbone pretreatment (Figures 5(c), 6(c)), but increase in the cytoplasm. Moreover, negative control showed no immunoreactivity (Figures 5(d), 6(d)).

### 3.5. NF-*κ*B p65 and I-*κ*B*α* Expression

**Figure 7:**
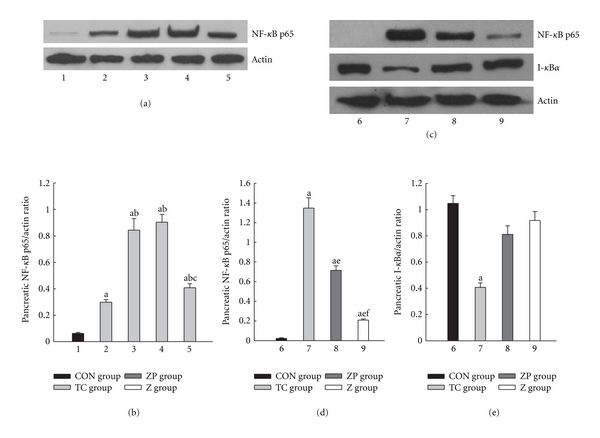
Expression of NF-*κ*B p65 and I-*κ*B*α* in pancreatic tissue. 1, 6 CON group; 2, TC group 1 h; 3, TC group 3 h; 4, TC group 6 h; 5, 7 TC group 12 h; 8, ZP group; 9, Z group. ^a^ *P* < 0.05 versus CON group. ^b^ *P* < 0.05 versus TC group 1 h. ^c^ *P* < 0.05 versus TC group 3 h. ^d^ *P* < 0.05 versus TC group 6 h. ^e^ *P* < 0.05 versus TC group 12 h. ^f^ *P* < 0.05 versus ZP group.

Figures 7(a)-7(b) showed peak expression of NF-*κ*B p65 protein in pancreas occurred at 3 h, duration to 6 h, decreased at 12 h in the TC group, which was higher than in the CON group. In the ZP group, the NF-*κ*B p65 expression declined to a much lower level than in the TC group (*P* < 0.05) (Figures 7(c)-7(d)). Moreover, only TC group showed a significant decrease of I-*κ*B*α* expression (Figure 7(e)).

**Figure 8:**
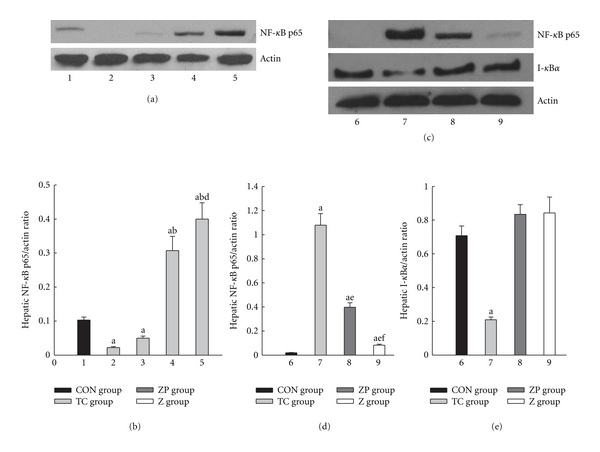
Expression of NF-*κ*B p65 and I-*κ*B*α* in hepatic tissue. 1, 6 CON group; 2, TC group 1 h; 3, TC group 3 h; 4, TC group 6 h; 5, 7 TC group 12 h; 8, ZP group; 9, Z group. ^a^ *P* < 0.05 versus CON group. ^b^ *P* < 0.05 versus TC group 1 h. ^c^ *P* < 0.05  versus TC group 3 h. ^d^ *P* < 0.05 versus TC group 6 h. ^e^ *P* < 0.05 versus TC group 12 h. ^f^ *P* < 0.05 versus ZP group.

Hepatic NF-*κ*B expression increased gradually after induction of pancreatitis in the TC group and the peak expression occurred at 12 h (Figures 8(a)-8(b)). Figures 8(c)-8(d) showed that the expression of NF-*κ*B p65 protein in the ZP group was lower than in the TC group. Furthermore, only TC group showed a significant decrease of hepatic I-*κ*B*α* expression (Figure 8(e)).

### 3.6. ICAM-1 and IL-1*β* mRNA Expression

**Figure 9:**
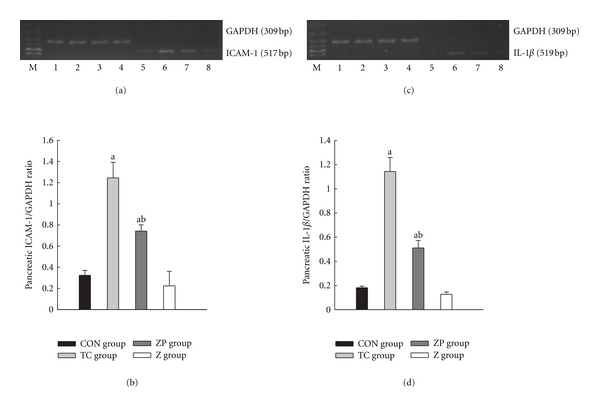
Expression of ICAM-1 and IL-1*β* mRNA in pancreatic tissue by RT-PCR analysis. 1–4 GAPDH; 5, CON group; 6, TC group 12 h; 7, ZP group; 8, Z group. ^a^ *P* < 0.05 versus CON group. ^b^ *P* < 0.05 versus TC group 12 h.

**Figure 10:**
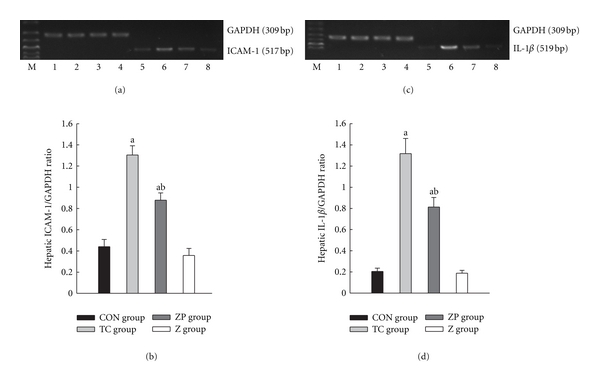
Expression of ICAM-1 and IL-1*β* mRNA in hepatic tissue by RT-PCR analysis. 1–4 GAPDH; 5, CON group; 6, TC group 12 h; 7, ZP group; 8, Z group. ^a^ *P* < 0.05 versus CON group. ^b^ *P* < 0.05 versus TC group 12 h.

In this study, expressions of ICAM-1 and IL-1*β* in pancreas were found to be much higher than in the CON group (*P* < 0.05) (Figures 9(a)–9(d)). However, these mRNA expressions were much lower in the ZP group than in the TC group (*P* < 0.05) (Figures 9(a)– 9(d)). The tendencies of these mRNA expressions in hepatic tissue (Figures 10(a)–10(d)) were the same as in pancreas.

## 4. Discussion

Zerumbone, as a component of a wild ginger, was first isolated and structurally elucidated forty years ago [22]. It has been widely used in experiments by virtue of its anti-neoplasms and anti-inflammatory properties [13, 15]. This study was to discuss the alleviating effect of zerumbone on hepatic injury following acute necrotizing pancreatitis.

In the preliminary study, the 12 h time point was chosen after pancreatitis was induced when intrapancreatic damage had already peaked during pancreatitis [20, 23]. It indicated that all concentrations of zerumbone injected via femoral vein had the ability to attenuate serum AMY. However, only 10 and 20 mg/kg zerumbone could decrease serum sPLA2 and ALT. Therefore, the minimum effective dose (optimal dose) was 10 mg/kg. It had been reported that doses of 0.1, 1, 10, 20, and 100 mg/kg zerumbone were tested on Wistar rats via intraperitoneal injection following cholecystokinin octapeptide-induced pancreatitis. The dose of 20 mg/kg zerumbone was the optimal dose [18]. Another study used male ICR mice, receiving intraperitoneal injection of zerumbone (5, 10, 50, and 100 mg/kg) to estimate the effect of zerumbone on paw edema, granuloma, and toxicity test. It was demonstrated that all zerumbone doses could suppress granulomatous tissue formation in cotton pellet-induced granuloma test [24].

The increased AMY and sPLA_2_ levels in the TC group and the elevated histopathological score of pancreas [25] indicated that pancreas injury deteriorated gradually following ANP. Moreover, the increased AMY and sPLA_2_ levels advocated the theory of autodigestion of pancreas. [26]. In the TC group, increased ALT and AST levels and hepatic histological examination also showed the damage of hepatic injury from bad to worse [27]. Is there any relationship between the changes in pancreatic and hepatic tissues?

Previous researches showed that the active RelA/p65 NF-*κ*B subunit played an essential role in systemic inflammatory response in pancreatitis [28–30]. Cytoplasmic I*κ*B proteins were primary regulators interacting with NF-*κ*B subunits in the cytoplasm of normal cells [31–33]. Upon stimulation, these I*κ*B proteins were rapidly degraded, allowing NF-*κ*B translocate into nucleus and activate the transcription of related genes: ICAM-1 and IL-1*β* [34–36]. Other studies showed that downregulation of NF-*κ*B not only inhibited ICAM-1 and IL-1*β* expression, but also attenuated pancreatic and hepatic injury in pancreatitis [35–37].

The result of immunohistochemisty assay indicated that NF-*κ*B has been shown to translocate from the cytoplasm into the nucleus upon activation in pancreatic or hepatic tissues during pancreatitis. The western blot results showed that the I-*κ*B*α* protein expression was much lower during pancreatitis because of rapidly degradation. Pancreatic NF-*κ*B expression elevated from 1 h, peaked at 3 h and continued to 6 h following ANP. It is accordance with published data [38]. The peaking expression of NF-*κ*B in hepatic tissue was lagged behind up to 12 h following ANP. These results supported that the early phase of NF-*κ*B activation started from pancreas by translocating into the nucleus. Up to 12 h in ANP, the NF-*κ*B expression in hepatic tissue was strong due to the inflammatory cascade. The multiple organ dysfunction syndromes may occur relying on the NF-*κ*B inflammatory cascade, if longer or more organs were applied. However, the peaking expression of NF-*κ*B in hepatic tissue lagged behind in pancreas, thus the period from 3 h to 12 h following ANP was defined as “window period,” which was the potential period for hepatic protection.

It was reported that zerumbone exerted a beneficial effect on inflammation but failed to improve histology [18]. Furthermore, published data indicated that administration of zerumbone could decrease NF-*κ*B p65 expression [14, 39–41]. Triptolide as well as zerumbone, was a crude plant extract and could attenuate hepatic injury and inhibit NF-*κ*B activation [42]. Western blot and immunohistochemisty assay in this study showed that zerumbone inhibited NF-*κ*B activation and decreased ICAM-1 and IL-1*β* mRNA expression. The serum assay and the changes in histopathological examination indicated that zerumbone attenuated the severity of acute necrotizing pancreatitis and pancreatitis-induced hepatic injury.

In summary, this work suggests that zerumbone attenuates the severity of acute necrotizing pancreatitis and pancreatitis-induced hepatic injury, through inhibiting NF-*κ*B activation and downregulation of intercellular adhesion molecule-1 and Interleukin-1*β*. However, its complete molecular mechanism still remains unclear. These works can serve as a basis for further studies on the therapeutic potential of zerumbone in acute necrotizing pancreatitis.

## Figures and Tables

**Table 1:** Experimental design of the preliminary research.

Group	*n*	Time		
		−0.5 h	0 h	12 h
Sham-operated group	8	Vehicle (10% DMSO)	Saline	Sacrifice
Acute necrotizing pancreatitis group	8	Vehicle (10% DMSO)	Taurocholate	Sacrifice
Zerumbone pretreated group 5 mg/kg	8	Zerumbone 5 mg/kg	Taurocholate	Sacrifice
Zerumbone pretreated group 10 mg/kg	8	Zerumbone 10 mg/kg	Taurocholate	Sacrifice
Zerumbone pretreated group 20 mg/kg	8	Zerumbone 20 mg/kg	Taurocholate	Sacrifice
Zerumbone pretreated group 40 mg/kg	8	Zerumbone 40 mg/kg	Taurocholate	Sacrifice

In acute necrotizing pancreatitis group, rats received the vehicle (10% DMSO, 2 mL/kg) administered via femoral vein half an hour prior to retrograde infusion of a freshly prepared 5% sodium taurocholate solution (1 mg/kg). In sham-operated group, rats received the vehicle administered via femoral vein and after half an hour, the rats were retrograde infused isotonic saline solution (1 mL/kg). In zerumbone pretreated group, rats received 5, 10, 20, and 40 mg/kg zerumbone dissolved in vehicle (10% DMSO v/v) administered via femoral vein half an hour prior to infusion 5% sodium taurocholate solution (1 mg/kg).

**Table 2:** Experimental design of the formal study.

Group	Time					
	−0.5 h	0 h	1 h	3 h	6 h	12 h
CON	Vehicle (10% DMSO)	Saline	Sacrifice (*n* = 16)	Sacrifice (*n* = 16)	Sacrifice (*n* = 16)	Sacrifice (*n* = 16)
TC	Vehicle (10% DMSO)	Taurocholate	Sacrifice (*n* = 16)	Sacrifice (*n* = 16)	Sacrifice (*n* = 16)	Sacrifice (*n* = 16)
ZP	Zerumbone 10 mg/kg	Taurocholate				Sacrifice (*n* = 16)
Z	Zerumbone 10 mg/kg	Saline				Sacrifice (*n* = 16)

In the TC group, rats received the vehicle (10% DMSO, 2 mL/kg) administered via femoral vein half an hour prior to retrograde infusion of a freshly prepared 5% sodium taurocholate solution (1 mg/kg). In the CON group, rats received the vehicle administered via femoral vein. After half an hour, the rats were retrograde infused isotonic saline solution (1 mL/kg). In the ZP group, rats received 10 mg/kg zerumbone dissolved in vehicle (10% DMSO v/v) administered via femoral vein half an hour prior to infusion 5% sodium taurocholate solution (1 mg/kg). In the Z group, rats received the same as ZP group except infusion isotonic saline solution instead of taurocholate.

**Table 3:** Preliminary study results of different doses of zerumbone.

Group	*n*	AMY (U/L)	sPLA2 (U/L)	ALT (U/L)
Sham-operated group	8	1304.8 ± 114.7	519.7 ± 63.9	95.7 ± 7.9
Acute necrotizing pancreatitis group	8	6774.8 ± 739.0^a^	789.0 ± 124.2^a^	257.6 ± 41.7^a^
Zerumbone pretreated group 5 mg/kg	8	5279.5 ± 857.4^a,b^	775.5 ± 160.9^a^	230.1 ± 50.4^a^
Zerumbone pretreated group 10 mg/kg	8	3342.6 ± 542.4^a,b,c^	621.7 ± 73.8	157.5 ± 31.2^a,b^
Zerumbone pretreated group 20 mg/kg	8	3440.9 ± 575.9^a,b,c^	625.9 ± 108.9	181.7 ± 30.3^a,b^
Zerumbone pretreated group 40 mg/kg	8	3073.7 ± 461.5^a,b,c^	667.0 ± 108.2^a^	245.8 ± 30.5^a^

Rats from different groups were ended at 12 h after sodium taurocholate infusion. The serum amylase (AMY), secretory phospholipase A_2_ (sPLA_2_), and alanine aminotransferase (ALT) in serum was measured by an automatic biochemistry analyzer with standard techniques (Olympus Optical Ltd., Japan). Data were expressed as means ± standard error values. ^a^ *P* < 0.05 versus sham-operated group. ^b^ *P* < 0.05 versus acute necrotizing pancreatitis group. ^c^ *P* < 0.05 versus zerumbone pretreated group 5 mg/kg.

**Table 4:** Hepatic pathological grades with sodium taurocholate-induced pancreatitis.

Group	*n*	Grade				Mean rank
		0	1	2	3	
CON group	12	12	0	0	0	10.5
TC group	12	0	1	1	10	40.7^a^
ZP group	12	0	4	6	2	31.5^ab^
Z group	12	8	3	1	0	15.8

Rats from different groups were ended at 12 h after sodium taurocholate infusion. The sections were evaluated by two independent pathologists who were blinded to this research. The change of hepatic sections was detected by Camargo's description [21]. Each value is the number of animals with grading changes. ^a^ *P* < 0.05 versus CON group. ^b^ *P* < 0.05 versus TC group.

## References

[1] Mitchell RMS, Byrne MF, Baillie J (2003). Pancreatitis. *The Lancet*.

[2] Vaquero E, Gukovsky I, Zaninovic V, Gukovskaya AS, Pandol SJ (2001). Localized pancreatic NF-*κ*B activation and inflammatory response in taurocholate-induced pancreatitis. *American Journal of Physiology*.

[3] Yang R, Shaufl AL, Killeen ME, Fink MP (2009). Ethyl pyruvate ameliorates liver injury secondary to severe acute pancreatitis. *Journal of Surgical Research*.

[4] Hori Y, Takeyama Y, Ueda T, Shinkai M, Takase K, Kuroda Y (2000). Macrophage-derived transforming growth factor-*β*1 induces hepatocellular injury via apoptosis in rat severe acute pancreatitis. *Surgery*.

[5] Dobosz M, Hac S, Mionskowska L, Dymecki D, Dobrowolski S, Wajda Z (2005). Organ microcirculatory disturbances in experimental acute pancreatitis. A role of nitric oxide. *Physiological Research*.

[6] Shen Y, Cui N, Miao B, Zhao E (2011). Immune dysregulation in patients with severe acute pancreatitis. *Inflammation*.

[7] Kitayama T, Nagao R, Masuda T (2002). The chemistry of zerumbone IV: asymmetric synthesis of Zerumbol. *Journal of Molecular Catalysis B*.

[8] Sung B, Jhurani S, Ahn KS (2008). Zerumbone down-regulates chemokine receptor CXCR4 expression leading to inhibition of CXCL12-induced invasion of breast and pancreatic tumor cells. *Cancer Research*.

[9] Kim M, Miyamoto S, Yasui Y, Oyama T, Murakami A, Tanaka T (2009). Zerumbone, a tropical ginger sesquiterpene, inhibits colon and lung carcinogenesis in mice. *International Journal of Cancer*.

[10] Murakami A, Miyamoto M, Ohigashi H (2004). Zerumbone, an anti-inflammatory phytochemical, induces expression of proinflammatory cytokine genes in human colon adenocarcinoma cell lines. *Biofactors*.

[11] Murakami A, Tanaka T, Lee JY (2004). Zerumbone, a sesquiterpene in subtropical ginger, suppresses skin tumor initiation and promotion stages in ICR mice. *International Journal of Cancer*.

[12] Nakamura Y, Yoshida C, Murakami A, Ohigashi H, Osawa T, Uchida K (2004). Zerumbone, a tropical ginger sesquiterpene, activates phase II drug metabolizing enzymes. *FEBS Letters*.

[13] Murakami A, Hayashi R, Tanaka T, Kwon KH, Ohigashi H, Safitri R (2004). Erratum: suppression of dextran sodium sulfate-induced colitis in mice by zerumbone, a subtropical ginger sesquiterpene, and nimesulide: separately and in combination. *Biochemical Pharmacology*.

[14] Murakami A, Matsumoto K, Koshimizu K, Ohigashi H (2003). Effects of selected food factors with chemopreventive properties on combined lipopolysaccharide-and interferon-*γ*-induced I*κ*B degradation in RAW264.7 macrophages. *Cancer Letters*.

[15] Chien TY, Chen LG, Lee CJ, Lee FY, Wang CC (2008). Anti-inflammatory constituents of Zingiber zerumbet. *Food Chemistry*.

[16] Murakami A, Takahashi D, Koshimizu K, Ohigashi H (2003). Synergistic suppression of superoxide and nitric oxide generation from inflammatory cells by combined food factors. *Mutation Research*.

[17] Murakami A, Takahashi D, Kinoshita T (2002). Zerumbone, a Southeast Asian ginger sesquiterpene, markedly suppresses free radical generation, proinflammatory protein production, and cancer cell proliferation accompanied by apoptosis: The *α*,*β*-unsaturated carbonyl group is a prerequisite. *Carcinogenesis*.

[18] Annamaria S, Laszlo T, Jozsef K (2007). Zerumbone exerts a beneficial effect on inflammatory parameters of cholecystokinin octapeptide-induced experimental pancreatitis but fails to improve histology. *Pancreas*.

[19] Schmidt J, Rattner DW, Lewandrowski K (1992). A better model of acute pancreatitis for evaluating therapy. *Annals of Surgery*.

[20] Paszkowski AS, Rau B, Mayer JM, Moller P, Beger HG (2002). Therapeutic application of caspase 1/interleukin-1*β*-converting enzyme inhibitor decreases the death rate in severe acute experimental pancreatitis. *Annals of Surgery*.

[21] Camargo CA, Madden JF, Gao W, Selvan RS, Clavien PA (1997). Interleukin-6 protects liver against warm ischemia/reperfusion injury and promotes hepatocyte proliferation in the rodent. *Hepatology*.

[22] Kitayama T, Okamoto T, Hill RK (1999). Chemistry of zerumbone—1. Simplified isolation, conjugate addition reactions, and a unique ring contracting transannular reaction of its dibromide. *Journal of Organic Chemistry*.

[23] Xia SH, Fang DC, Hu CX, Bi HY, Yang YZ, Di Y (2007). Effect of BN52021 on NF*κ*-Bp65 expression in pancreatic tissues of rats with severe acute pancreatitis. *World Journal of Gastroenterology*.

[24] Sulaiman MR, Perimal EK, Akhtar MN (2010). Anti-inflammatory effect of zerumbone on acute and chronic inflammation models in mice. *Fitoterapia*.

[25] Xu S, Chen C, Wang WX, Huang SR, Yu J, Chen XY (2010). Crucial role of group IIA phospholipase A2 in pancreatitis-associated adrenal injury in acute necrotizing pancreatitis. *Pathology Research and Practice*.

[26] Kylanpaa ML, Repo H, Puolakkainen PA (2010). Inflammation and immunosuppression in severe acute pancreatitis. *World Journal of Gastroenterology*.

[27] Zhang XP, Zhang L, Wang Y (2007). Study of the protective effests of dexamethasone on multiple organ injury in rats with severe acute pancreatitis. *Journal of the Pancreas*.

[28] Telek G, Ducroc R, Scoazec JY, Pasquier C, Feldmann G, Roze C (2001). Differential upregulation of cellular adhesion molecules at the sites of oxidative stress in experimental acute pancreatitis. *Journal of Surgical Research*.

[29] Chen X, Ji B, Han B, Ernst SA, Simeone D, Logsdon CD (2002). NF-*κ*B activation in pancreas induces pancreatic and systemic inflammatory response. *Gastroenterology*.

[30] Peng Y, Gallagher SF, Haines K, Baksh K, Murr MM (2006). Nuclear factor-*κ*B mediates Kupffer cell apoptosis through transcriptional activation of Fas/FasL. *Journal of Surgical Research*.

[31] Ding J, Song D, Ye X, Liu SF (2009). A pivotal role of endothelial-specific NF-*κ*B signaling in the pathogenesis of septic shock and septic vascular dysfunction. *Journal of Immunology*.

[32] Lee HJ, Lim HJ, Lee DY (2010). Carabrol suppresses LPS-induced nitric oxide synthase expression by inactivation of p38 and JNK via inhibition of I-*κ*B*α* degradation in RAW 264.7 cells. *Biochemical and Biophysical Research Communications*.

[33] Xu H, Ye X, Steinberg H, Liu SF (2010). Selective blockade of endothelial NF-*κ*B pathway differentially affects systemic inflammation and multiple organ dysfunction and injury in septic mice. *Journal of Pathology*.

[34] Manavalan B, Basith S, Choi YM, Lee G, Choi S (2010). Structure-function relationship of cytoplasmic and nuclear I*κ*B proteins: an in silico analysis. *PLoS ONE*.

[35] Xiping Z, Dijiong W, Jianfeng L (2009). Effects of salvia miltiorrhizae on ICAM-1, TLR4, NF-*κ*B and bax proteins expression in multiple organs of rats with severe acute pancreatitis or obstructive jaundice. *Inflammation*.

[36] Xu MQ, Shuai XR, Yan ML, Zhang MM, Yan LN (2005). Nuclear factor-*κ*B decoy oligodeoxynucleotides attenuates ischemia/reperfusion injury in rat liver graft. *World Journal of Gastroenterology*.

[37] Li YY, Li XL, Yang CX, Zhong H, Yao H, Zhu L (2003). Effects of tetrandrine and QYT on ICAM-1 and SOD gene expression in pancreas and liver of rats with acute pancreatitis. *World Journal of Gastroenterology*.

[38] Gukovsky I, Gukovskaya AS, Blinman TA, Zaninovic V, Pandol SJ (1998). Early NF-*κ*B activation is associated with hormone-induced pancreatitis. *American Journal of Physiology*.

[39] Sung B, Murakami A, Oyajobi BO, Aggarwal BB (2009). Zerumbone abolishes RaNKL-induced NF-*κ*B activation, inhibits osteoclastogenesis, and suppresses human breast cancer-induced bone loss in athymic nude mice. *Cancer Research*.

[40] Takada Y, Murakami A, Aggarwal BB (2005). Zerumbone abolishes NF-*κ*B and I*κ*B*α* kinase activation leading to suppression of antiapoptotic and metastatic gene expression, upregulation of apoptosis, and downregulation of invasion. *Oncogene*.

[41] Aggarwal BB, Kunnumakkara AB, Harikumar KB, Tharakan ST, Sung B, Anand P (2008). Potential of spice-derived phytochemicals for cancer prevention. *Planta Medica*.

[42] Zhao YF, Zhai WL, Zhang SJ, Chen XP (2005). Protection effect of triplolide to liver injury in rats with severe acute pancreatitis. *Hepatobiliary and Pancreatic Diseases International*.

